# Early hypertension and neutropenia are predictors of treatment efficacy in metastatic colorectal cancer patients administered FOLFIRI and vascular endothelial growth factor inhibitors as second‐line chemotherapy

**DOI:** 10.1002/cam4.3638

**Published:** 2020-12-21

**Authors:** Hiroki Osumi, Eiji Shinozaki, Akira Ooki, Takeru Wakatsuki, Daisaku Kamiimabeppu, Taro Sato, Izuma Nakayama, Mariko Ogura, Daisuke Takahari, Keisho Chin, Kensei Yamaguchi

**Affiliations:** ^1^ Department of Gastroenterology Cancer Institute Hospital Japanese Foundation for Cancer Research Tokyo Japan

**Keywords:** angiogenesis inhibitors, colorectal cancer, drug response biomarkers, vascular endothelial growth factors

## Abstract

**Background:**

Three vascular endothelial growth factor (VEGF) inhibitors, Bevacizumab (BEV), ramucirumab (RAM), and aflibercept (AFL), are widely used for metastatic colorectal cancer (mCRC) patients who are treated with second‐line chemotherapy. The difference in outcome between the three drugs has not been evaluated. In contrast to epidermal growth factor receptor inhibitors, VEGF inhibitors have few candidate predictors of efficacy.

**Methods:**

Consecutive mCRC patients who were treated with second‐line chemotherapy were retrospectively enrolled. Overall response rate (ORR), progression‐free survival (PFS), overall survival (OS), and safety were assessed. Subgroup analyses of prognostic and predictive efficacy markers were performed.

**Results:**

A total of 119 (41.2%), 107 (37.0%), and 63 patients (21.8%) were treated with FOLFIRI +BEV, RAM, or AFL, respectively. ORR, PFS, and OS showed no significant differences between three groups. However, the frequency of grade 3 or 4 adverse events (AEs) in the FOLFIRI +AFL group was significantly higher than that in the other groups (*p* < 0.001). Patients with grade 3 or 4 AEs, especially hypertension and neutropenia within the first four cycles of treatment had significantly longer PFS and OS than those without AEs, irrespective of treatment with VEGF inhibitors (*p* < 0.001). PFS in patients without prior BEV exposure was also significantly longer than that in patients with prior BEV exposure (*p* = 0.003).

**Conclusions:**

Chemotherapeutic efficacy did not differ between the groups. Grade 3 or 4 AEs within the first four cycles of treatment and prior BEV exposure may be an effective predictor of treatment efficacy in mCRC patients administered VEGF inhibitors as second‐line chemotherapy.

## Graphical abstract

Chemotherapeutic efficacy did not differ between the groups. Grade 3 or 4 AEs within the first four cycles of treatment may be an effective predictor of treatment efficacy in mCRC patients administered VEGF inhibitors as second‐line chemotherapy

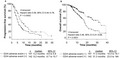

## 1 INTRODUCTION

Angiogenesis is induced by the release of angiogenic factors in tissues with ischemia, such as cancer or wound healing, due to hypoxia and growth factors.^1^ One of the glycoproteins involved in angiogenesis is vascular endothelial growth factor (VEGF) and excessive secretion of VEGF in cancer leads to abnormal angiogenesis.^2^, ^3^ Since tumors are accompanied by abnormal vascular structure, tumor fluid interstitial pressure increases due to vascular leakage and is accompanied by hypoxia.^4^, ^5^ These factors are related to tumor progression and treatment resistance.^6^, ^7^ Antiangiogenic drugs can inhibit the supply of oxygen and nutrients to the cancer, therefore suppressing the growth of the cancer. In addition, by reducing vascular permeability and normalizing interstitial pressure, concomitant cytotoxic chemotherapy can be delivered to cancer more easily.^8^ Combination treatment of antiangiogenic drugs and cytotoxic chemotherapy are recommended for metastatic colorectal cancer (mCRC) patients as a second‐line chemotherapy.^9^, ^10^ because the addition of these drugs significantly increases the overall survival (OS) compared to cytotoxic chemotherapy alone.^11^, ^12^, ^13^ Bevacizumab (BEV) is a monoclonal antibody against VEGF, which inhibits the action of VEGF, therefore suppressing angiogenesis and tumor growth and metastasis.^14^ The phase III ML18147 trial reported BEV improves survival in patients who had already received BEV as first‐line therapy (HR 0.83, *p* = 0.021).^11^ Ramucirumab (RAM), an anti‐VEGF receptor 2 (VEGFR‐2) fully human monoclonal IgG1 antibody, is a member of the antibody class of molecularly targeted therapies that works to inhibit tumor growth by preventing VEGF from binding to VEGFR‐2 and sending angiogenic signals downstream.^15^ The phase III RAISE trial showed a significantly survival benefit for patients who were treated with RAM +FOLFIRI (HR 0.84, *p* = 0.0219).^16^ Aflibercept (AFL) is a recombinant fusion protein consisting of the extracellular domain of the human VEGF receptor 1 and 2 proteins and the Fc portion of the human antibody IgG1.^17^, ^18^ The phase III VELOUR study revealed that compared to the placebo, AFL addition resulted in a significantly increased survival rate (HR 0.817, *p* = 0.0032).^13^ According to these results, these antiangiogenic drugs were approved in Japan as combination therapy with FOLFIRI in a second‐line setting.^10^ However, at present, there is no randomized trial directly comparing the three antiangiogenic drugs (BEV, RAM, and AFL) with FOLFIRI in second‐line mCRC treatment. Furthermore, there are only a few reports about predictive and/or surrogate biomarkers of treatment efficacy for second‐line VEGF inhibitor containing chemotherapy^12^, ^19^ although there is several reports that hypertension may be surrogate marker of clinical outcome of first‐line chemotherapy with BEV in mCRC.^20^, ^21^ The present study evaluated both the efficacy and safety among mCRC patients who were treated with FOLFIRI +BEV, RAM, or AFL as second‐line chemotherapy, and explored the predictive biomarkers for treatment efficacy to contribute information required for appropriate clinical decision‐making processes.

## 2 MATERIALS AND METHODS

### 2.1 Patients and treatment schedule

Two hundred and eighty‐nine mCRC patients who were treated with second‐line chemotherapies at our hospital, from January 2017 to December 2019 were retrospectively enrolled in the current study. BEV was administered at the recommended dose of 5 mg/kg. RAM was administered at the recommended dose of 8 mg/kg. AFL was administered at the recommended dose of 4 mg/kg. The concomitant chemotherapy was FOLFIRI (irinotecan 150–180 mg/m^2^, L‐leucovorin 200 mg/m^2^, bolus 5‐FU 400 mg/ m^2^, 46‐h infusion of 5‐FU 2,400 mg/ m^2^). Prophylactic treatments and dose reduction were performed based on recommendations of guidelines and physician's decisions.

### 2.2 Assessments

We collected the data identified by medical record and/or imaging. We confirmed age, sex, primary site, metastatic site, *RAS* status in tissue, prior BEV exposure in first‐line chemotherapy, first‐line progression‐free survival (patients treated with BEV only), patients who experienced relapse within 6 months of completing oxaliplatin‐based adjuvant therapy, and tumor markers (CEA and CA19‐9). Complete response (CR), partial response (PR), stable disease (SD), and progressive disease (PD) were defined based on RECIST guidelines, v1.1. Objective response rate (ORR) denoted the proportion of patients who had a CR or PR to second‐line chemotherapy, and disease control rate (DCR) indicated the proportion of patients who had a CR, PR, or SD response to therapy. We defined progression‐free survival (PFS) as the time from the first day of second‐line treatment to either the first objective evidence of disease progression or death from any cause. We also defined OS as the time from the first day of second‐line treatment until the time of death. We assessed the grade of adverse events (AEs) using the Common Toxicity Criteria for Adverse Events (CTCAE) v4.0.

### 2.3 Statistical analyses

We estimated PFS and OS using the Kaplan–Meier method and also assessed the statistical significance of the correlation between the clinical outcome and clinical parameters using the log‐rank test. The t‐test, chi‐squared test, and Cox proportional hazard analysis were used for statistics tests. A value of *p* < 0.05 was considered statistically significant. In the Cox proportional hazard analysis, factors with *p* < 0.05 in the univariate analysis were included in the multivariate analysis (backward stepwise methods). Statistical analyses were performed using the EZR statistical software 1.41.^22^

## 3 RESULTS

### 3.1 Patient characteristics

The characteristics of 289 mCRC patients who were treated with second‐line chemotherapy in our hospital are shown in Table 1. Median age was 63.0 years (range, 31.0–84.0 years). A total of 119 (41.2%), 107 (37.0%), and 63 patients (21.8%) were treated with FOLFIRI +BEV, RAM, and AFL, respectively. No significant differences were observed about *RAS* status, location of primary tumor, or the ratio of BEV exposure in pretreatment among the three groups.

**TABLE 1:** Patient demographics and clinical characteristics.

Characteristics	Total (N = 289) No. of patients (%)	FOLFIRI + Bevacizumab (N = 119)	FOLFIRI + Ramucirumab (N = 107)	FOLFIRI + Aflibercept (N = 63)	*p* value
Age at enrollment, years					
Median [range]	63.0 [31.0–84.0]	63.0 [32.0–82.0]	64.0 [31–84.0]	62.0 [43.0–80.0]	0.87
Sex					
Male	137 (47.4)	57 (47.9)	46 (43.0)	34 (54.0)	0.39
Female	152 (52.6)	62 (52.1)	61 (57.0)	29 (46.0)	
Primary site					
Right‐sided colon	93 (32.2)	39 (32.8)	22 (20.6)	17 (27.0)	0.12
Left‐sided colon	196 (67.8)	80 (67.2)	85 (79.4)	46 (73.0)	
Metastatic site					
Liver	148 (51.2)	58 (48.7)	59 (55.1)	31 (49.2)	0.59
Lung	145 (50.2)	66 (55.5)	49 (45.8)	36 (57.1)	0.25
Peritoneal	97 (33.6)	38 (31.9)	31 (29.0)	25 (39.7)	0.34
Lymph node	99 (34.3)	40 (33.6)	40 (37.4)	17 (27.0)	0.38
Other	41 (14.2)	14 (11.8)	16 (15.0)	11 (17.5)	0.54
*RAS* status in tissue					
Wild type	134 (46.4)	47 (39.5)	57 (53.3)	30 (47.6)	0.11
Mutant	155 (53.6)	72 (60.5)	50 (46.7)	33 (52.4)	
Prior bevacizumab exposure in first‐line chemotherapy					
Yes	159 (55.0)	68 (57.1)	54 (50.5)	37 (58.7)	0.55
No	130 (45.0)	51 (42.9)	53 (49.5)	26 (41.3)	
First‐line progression‐free survival (Patients treated with Bevacizumab only)					
≤9 months	71 (44.7)	25 (36.8)	29 (53.7)	17 (45.9)	0.16
>9 months	88 (55.3)	43 (63.2)	25 (46.3)	20 (54.1)	
Patients who experienced relapse within 6 months of completing oxaliplatin‐based adjuvant therapy					
Yes	40 (13.8)	15 (12.6)	15 (14.0)	10 (15.9)	0.84
No	249 (86.2)	104 (87.4)	92 (86.0)	53 (84.1)	
Tumor markers (at initiation of second‐line chemotherapy)					
CEA median, [range]	17.3 [0.5–17056.1]	16.8 [1.0–1501.4]	28.3 [0.5–17056.1]	14.9 [1.0–7415]	0.30
CA19‐9 median, [range]	32.7 [2.0–50000]	27.6 [2.0–29210.2]	33.0 [2.0–50000]	33.6 [2.0–50000]	0.71
RAS:rat sarcoma viral oncogene homolog					
CEA: carcinoembryonic antigen					
CA19‐9: carbohydrate antigen 19–9					

### 3.2 Survival endpoints and factors associated with survival

**FIGURE 1:**
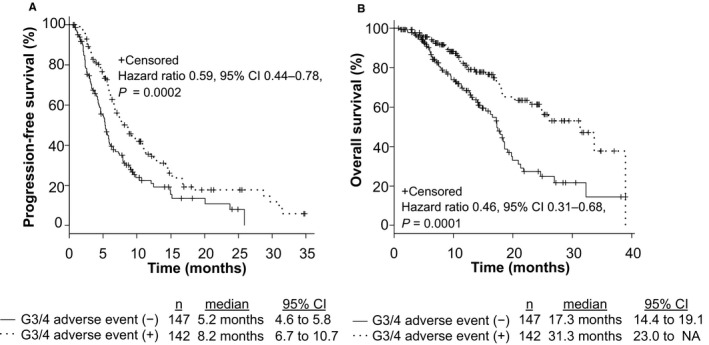
Progression‐free survival (PFS) and overall survival (OS) with respect to grade 3 or 4 adverse events (AEs) within the first four cycles of treatment in metastatic colorectal cancer (mCRC) patients who were treated with second‐line chemotherapy. This figure shows PFS (A) and OS (B) in patients with grade 3 or 4 AEs within the first four cycles of treatment compared to those without grade 3 or 4 AEs

**FIGURE 2:**
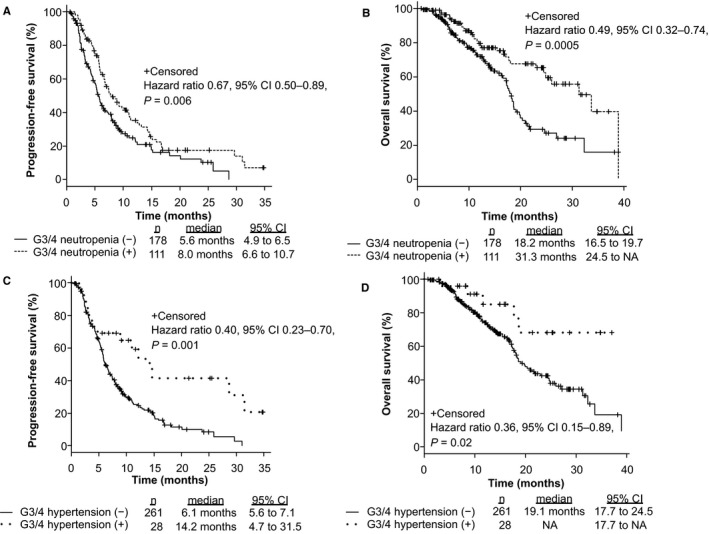
Progression‐free survival (PFS) and overall survival (OS) with respect to grade 3 or 4 neutropenia and hypertension within the first four cycles of treatment in mCRC patients who were treated with second‐line chemotherapy. This figure shows PFS (A, C) and OS (B, D) in patients with grade 3 or 4 neutropenia and hypertension within the first four cycles of treatment compared to those without grade 3 or 4 neutropenia and hypertension

**FIGURE 3:**
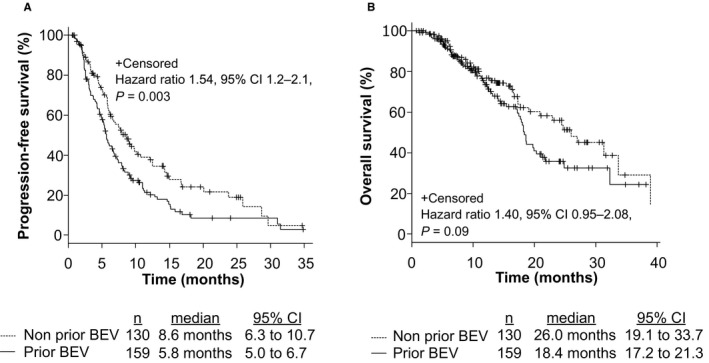
Progression‐free survival (PFS) and overall survival (OS) with respect to prior bevacizumab (BEV) exposure in mCRC patients who were treated with second‐line chemotherapy. This figure shows PFS (A) and OS (B) in patients with prior BEV exposure compared to those without prior BEV exposure

To assess the clinical efficacy of FOLFIRI +each antiangiogenic drug in mCRC patients, we compared PFS, OS, and ORR among patients treated with FOLFIRI +BEV, FOLFIRI +RAM, and FOLFIRI +AFL. The median PFS values were 7.2 months (6.0–9.0), 5.8 months (4.6–6.8), and 8.2 months (5.2–12.8), respectively (*p* = 0.21; Figure S1A). The median OS was 18.6 months (17.4–21.3), 23.0 months (16.7–31.3), and NA, respectively (*p* = 0.47; Figure S1B). The ORR from each group was 15.1%, 11.2%, and 17.4%, respectively (*p* = 0.48, Table S1). No significant differences were observed between the groups based on *RAS* status and primary tumor location (*RAS* status: *RAS* wild type vs. *RAS* mutant; 6.5 months vs. 6.7 months, *p* = 0.93; primary tumor location: left side vs. right side; 7.1 months vs. 6.0 months, *p* = 0.09). Patients with grade 3 or 4 AEs within the first four cycles of treatment were related to significantly longer PFS (*p* = 0.0002; Figure 1A) and OS (*p* = 0.0001; Figure 1B); the ORR from each group was 16.9% and 12.2%, respectively (*p* = 0.32). In analysis of each AE, treatment‐induced neutropenia and hypertension within the first four cycles of treatment were related to significantly longer PFS (Neutropenia: *p* = 0.006, Hypertension: *p* = 0.001; Figure 2A,C) and OS (Neutropenia: *p* = 0.0005, Hypertension: *p* = 0.02; Figure 2B,D). Figure S2 shows that grade 3 or 4 AEs within the first four cycles of treatment were related to significantly longer PFS in patients both with and without prior BEV exposure (Non‐prior BEV: *p* = 0.009; prior BEV: *p* = 0.00001). Mean dose intensities of irinotecan within the first four cycles were 61.2 mg/m^2^/week, 60.5 mg/m^2^/week, and 59.0 mg/m^2^/week, respectively (*p* = 0.76). PFS in non‐prior BEV patients was also significantly longer than prior BEV patients (*p* = 0.003; Figure 3A); the ORR from each group was 21.5% and 7.5%, respectively (*p* = 0.0009).

### 3.3 Toxicity

We next analyzed the AEs of mCRC patients who were treated with FOLFIRI +antiangiogenic drugs. Grade 3 or 4 AEs occurred in 168 patients (58.1%). AE occurrences in 168 patients with mCRC are summarized in Table 2. No treatment‐related death was observed. The most common grade 3 or 4 AEs were neutropenia (49.1%), hypertension (10.0%), and proteinuria (8.0%), respectively. Febrile neutropenia was observed in five patients (1.7%). The frequency of grade 3 or 4 AEs in the FOLFIRI +AFL group was significantly higher than those of the FOLFIRI +BEV group (any grade 3 or 4 AE_s_: 46.2%, 57.0% and 82.5%, *p* < 0.001; neutropenia: 38.7%, 56.1%, and 57.1%, *p* = 0.01; nausea: 0%, 0.9%, 4.8%, *p* = 0.02; hypertension: 1.7%, 11.2%, and 23.8%, *p* = 0.000007 in the FOLFIRI +BEV, RAM, and AFL groups, respectively).

**TABLE 2:** Incidence of grade 3 or 4 of adverse events according to second‐line chemotherapy

Adverse event	FOLFIRI + Bevacizumab (N = 119)	FOLFIRI + Ramucirumab (N = 107)	FOLFIRI + Aflibercept (N = 63)	*p* value
Neutropenia, (%)	46 (38.7)	60 (56.1)	36 (57.1)	0.01
Febrile neutropenia, (%)	1 (0.8)	3 (2.8)	1 (1.6)	0.64
Nausea, (%)	0 (0)	1 (0.9)	3 (4.8)	0.02
Diarrhea, (%)	5 (4.2)	5 (4.7)	5 (7.9)	0.55
Hypertension, (%)	2 (1.7)	12 (11.2)	15 (23.8)	0.000007
Thromboembolic events, (%)	4 (3.4)	2 (1.9)	1 (1.6)	0.79
Proteinuria, (%)	6 (5.0)	9 (8.4)	8 (12.7)	0.18
Bleeding, (%)	0 (0)	0 (0)	2 (3.2)	0.047
Mucotitis, (%)	2 (1.7)	2 (1.9)	1 (1.6)	1
Infection, (%)	1 (0.8)	2 (1.9)	1 (1.6)	0.83
Fatigue, (%)	4 (3.4)	2 (1.9)	0 (0)	0.38
Edema, (%)	1 (0.8)	2 (1.9)	0 (0)	0.61
Renal failure, (%)	0 (0)	1 (0.9)	1 (1.6)	0.35

The frequency of dose reduction or delayed treatment was 68.1% (81/119), 68.8% (75/109), and 68.3% (43/63) in the FOLFIRI +BEV, RAM, and AFL groups, respectively (*p* = 1). The relative dose intensity of each VEGF inhibitor was 72.7%, 72.1%, and 65.0%, respectively (*p* = 0.42), while the duration of administration of each VEGF inhibitor was 4.9 months, 4.4 months, and 5.2 months, respectively, in the same groups (*p* = 0.28). The frequency of grade 3 or 4 AEs within the first four cycles of treatment in mCRC patients treated with second‐line chemotherapy is summarized in Table 3. The most common grade 3 or 4 AEs within the first four cycles of treatment were neutropenia (38.4%), hypertension (9.7%), and proteinuria (6.9%) in the FOLFIRI +BEV, RAM, and AFL groups, respectively. The frequency of grade 3 or 4 AEs in the FOLFIRI +AFL group was significantly higher than that in the FOLFIRI +BEV group (any grade 3 or 4 AE_s_: 37.0%, 49.5%, and 71.4%, *p* = 0.00005; neutropenia: 26.9%, 43.9%, and 50.8%, *p* = 0.002; nausea: 0%, 0%, and 4.8%, *p* = 0.01; hypertension: 1.7%, 11.2%, and 22.2%, *p* = 0.00002 in the FOLFIRI +BEV, RAM, and AFL groups, respectively).

**TABLE 3:** Incidence of grade 3 or 4 adverse events within the first four cycles of treatment in patients with mCRC treated with second‐line chemotherapy

Adverse event	FOLFIRI + Bevacizumab (N = 119)	FOLFIRI + Ramucirumab (N = 107)	FOLFIRI + Aflibercept (N = 63)	*p* value
Neutropenia, (%)	32 (26.9)	47 (43.9)	32 (50.8)	0.002
Febrile neutropenia, (%)	1 (0.8)	2 (1.9)	1 (1.6)	0.83
Nausea, (%)	0 (0)	0 (0)	3 (4.8)	0.01
Diarrhea, (%)	5 (4.2)	2 (1.9)	5 (7.9)	0.17
Hypertension, (%)	2 (1.7)	12 (11.2)	14 (22.2)	0.00002
Thromboembolic events, (%)	1 (0.8)	1 (0.9)	1 (1.6)	1
Proteinuria, (%)	6 (5.0)	8 (7.5)	6 (9.5)	0.50
Bleeding, (%)	0 (0)	0 (0)	2 (3.2)	0.047
Mucotitis, (%)	1 (0.8)	2 (1.9)	1 (1.6)	0.83
Infection, (%)	0 (0)	2 (1.9)	1 (1.6)	0.33
Fatigue, (%)	4 (3.4)	2 (1.9)	0 (0)	0.38
Edema, (%)	0 (0)	1 (0.9)	0 (0)	0.59
Renal failure, (%)	0 (0)	0 (0)	1 (1.6)	0.22

### 3.4 Univariate and multivariate analyses

In the univariate Cox proportional hazard analysis, liver metastasis, prior BEV exposure, and grade 3 or 4 AEs within the first four cycles were predictors for PFS (Table 4). Similarly, liver metastasis and grade 3 or 4 AEs within the first four cycles were predictors for OS (Table 4). Moreover, all of these were independent predictors for PFS (liver metastasis: HR 1.47, 95% CI 1.10–1.97, *p* = 0.01; prior BEV exposure: HR 1.52, 95% CI 1.13–2.05, *p* = 0.006; AEs: HR 0.57, 95% CI 0.43–0.77, *p* = 0.0002; Table 4) and OS (liver metastasis: HR 2.34, 95% CI 1.55–3.54, *p* = 0.00006; AEs: HR 0.44, 95% CI 0.29–0.66, *p* = 0.00009; Table 4) in the multivariate analysis.

**TABLE 4:** Cox proportional hazard analysis for PFS and OS in mCRC patients treated with second‐line chemotherapy

PFS	Univariate analysis				Multivariate analysis				
	HR	Lower 95% CI	Upper 95% CI	*p* value		HR	Lower 95% CI	Upper 95% CI	*p* value
Sex (Female* or Male)	0.85	0.63	1.15	0.29					
Age (<65* or ≥65)	0.96	0.71	1.29	0.76					
Primary tumor location (Left* or Right)	1.35	0.99	1.86	0.06					
Liver metastasis (Negative* or Positive)	1.50	1.11	2.02	0.008		1.47	1.10	1.97	0.01
Lung metastasis (Negative* or Positive)	0.92	0.68	1.24	0.58					
Peritoneal metastasis (Negative* or Positive)	1.21	0.89	1.65	0.22					
Lymph node metastasis (Negative* or Positive)	1.15	0.84	1.57	0.37					
Tissue *RAS* mutation (Negative* or Positive)	0.91	0.67	1.23	0.54					
Prior bevacizumab exposure in first‐line chemotherapy (Negative* or Positive)	1.51	1.11	2.04	0.008		1.52	1.13	2.05	0.006
Grade 3 or 4 adverse events within the first four cycles of treatment (Negative* or Positive)	0.58	0.44	0.78	0.0003		0.57	0.43	0.77	0.0002
Treatment regimen (Bevacizumab or other*)	1.10	0.83	1.46	0.51					

OS	Univariate analysis				Multivariate analysis			
	HR	Lower 95% CI	Upper 95% CI	*p* value	HR	Lower 95% CI	Upper 95% CI	*p* value
Sex (Female* or Male)	1.04	0.70	1.55	0.85				
Age (<65* or ≥65)	0.93	0.62	1.39	0.73				
Primary tumor location (Left* or Right)	1.33	0.87	2.04	0.18				
Liver metastasis (Negative* or Positive)	2.26	1.45	3.44	0.0001	2.34	1.55	3.54	0.00006
Lung metastasis (Negative* or Positive)	0.87	0.58		0.51				
Peritoneal metastasis (Negative* or Positive)	0.92	0.60	1.42	0.72				
Tissue *RAS* mutation (Negative* or Positive)	1.19	0.78	1.82	0.41				
Prior bevacizumab exposure in first‐line chemotherapy (Negative* or Positive)	1.45	0.96	2.18	0.07				
Grade 3 or 4 adverse events within the first four cycles of treatment (Negative* or Positive)	0.44	0.29	0.67	0.0001	0.44	0.29	0.66	0.00009
Treatment regimen (Bevacizumab or other*)	0.93	0.63	1.36	0.69				

## 4 DISCUSSION

To the best of our knowledge, this is the first report to evaluate safety and efficacy among FOLFIRI combined with BEV, RAM, or AFL as second‐line chemotherapy treatments in mCRC patients. No significant difference in chemotherapeutic efficacy was observed among the three groups. However, the AE rate was significantly higher, especially in the FOLFIRI +AFL group than in the FOLFIRI +BEV group. Furthermore, grade 3 or 4 AEs within the first four cycles were a surrogate marker for both PFS and OS, while prior BEV exposure was a predictor for PFS.

At present, there are no established efficacy biomarkers for any antiangiogenic drugs in mCRC. In this study a strong correlation was observed between the AEs, especially neutropenia and hypertension, and treatment efficacy. A previous report showed an association between the grade 3 or 4 diarrhea after the first cycle of irinotecan and the disease control.^23^ This result suggests an association between antitumor effect and irinotecan exposure and/or its metabolites.^23^ Indeed, neutropenia and delayed diarrhea have been shown to be related to both irinotecan and SN‐38 AUCs.^24^ However, the correlation between those parameters and tumor response has not been clearly clarified^25^; further study will be needed to clear this hypothesis. Furthermore, AEs related to antiangiogenic drugs as a surrogate marker of efficacy have shown in other cancers. Previous reports showed that VEGF binding to the VEGF receptor caused the induction of endothelial cells to increase nitric oxide production, which leads to vasodilation and reduced blood pressure.^19^, ^26^ Thus, hypertension is associated with impaired angiogenesis and represents bypass signaling pathway blockade of angiogenesis, especially in patients treated with BEV in first‐line chemotherapy. Furthermore, because angiogenesis inhibitors block the VEGF signaling, which is essential for the survival and maintenance of normal vascular endothelium, AEs of VEGF inhibitor may be derived from disorder to normal blood vessels.^27^ Thus, several single nucleotide polymorphisms (SNPs) that relate to VEGF pathways or drug metabolism/transport may be related to the risk of VEGF inhibitor‐related hypertension.^28^, ^29^ Retrospective studies have reported an association between the development of grade 2 or 3 hypertension with BEV in first‐line mCRC treatment with regard to ORR and PFS.^21^ In addition, the development of hypertension during RAM or AFL treatment has been related to improved efficacy in advanced cancers.^19^, ^30^ Although these results demonstrate that the emergence of grade 3 or 4 AEs after second‐line chemotherapy could be a surrogate marker of treatment efficacy in mCRC patients, further prospective study or subanalysis using a prospective study cohort is needed to verify our hypothesis. Although these factors may contribute to predicting the efficacy of second‐line chemotherapy, they did not serve as biomarkers contributing to the proper use of the three antiangiogenic drugs. In this study, as there was similar chemotherapeutic efficacy among the three groups and promising outcome was observed in patients with hypertension and neutropenia after second‐line chemotherapy, detrimental effects could be expected in patients without hypertension or neutropenia who were treated with FOLFIRI+RAM or AFL. Therefore, it is important to identify pretreatment biomarkers derived from the host rather than the tumor (e.g., SNPs) to identify these groups, especially in patients treated with FOLFIRI+RAM or AFL.

Our study also showed a strong correlation between prior Bev exposure and treatment efficacy. Considering prior BEV exposure, there were no phase III trials to validate the additional effect of BEV or RAM to FOLFIRI alone because all patients were treated with chemotherapy +BEV as first‐line treatment in the ML18147^11^ and RAISE^16^ trials. The E3200 trial reported the additional effect of BEV(10 mg/m^2^/2 weekly) compared to FOLFOX4 alone^31^; the median PFS and OS for the group treated with FOLFOX4 + BEV were significantly longer than those of the group treated with FOLFOX4 alone (PFS: HR 0.61, *p* < 0.0001; OS: HR 0.75, *p* = 0.0011).^31^ Furthermore, the ORR was 22.7% and 8.6%, respectively (*p* < 0.0001).^31^ Suzuki et al. reported the clinical outcomes of second‐line FOLFIRI +RAM for mCRC patients by prior BEV exposure.^32^ In that study, the median PFS in BEV‐naive patients was longer than those of prior BEV patients; the response rates were 23.0% and 3.0%, respectively (*p* = 0.0286).^32^ Furthermore, subanalysis of the VELOUR trial showed response rate in non‐prior BEV patients better than prior BEV patients treated with FOLFIRI +AFL (HR = 0.79 vs. 0.86).^13^ These results suggest that an additional effect of combination treatment with antiangiogenic drugs can be expected, especially in BEV‐naive patients, regardless of antiangiogenic drug type.

To date, although phase III trial data have been reported in antiangiogenic drugs combined with FOLFIRI,^11^, ^13^, ^16^ the differences in the ratio of cytotoxic chemotherapy (oxaliplatin or irinotecan) and BEV exposure in prior chemotherapy among the three trials make interpretation and comparison of the results difficult. Furthermore, there are no randomized studies to select the best antiangiogenic drugs after the first‐line chemotherapy in mCRC patients. In the current study, no significant difference in chemotherapeutic efficacy was observed among the three groups, similar to the comparison of the phase III trial results. However, the frequency of AEs in the current study was higher, especially neutropenia in the FOLFIRI +RAM group and both hematological and nonhematological toxicity in the FOLFIRI +AFL group than in the FOLFIRI +BEV group. In particular, the frequency of severe neutropenia in the FOLFIRI +AFL group exceeded 50%. In a Japanese phase II study to evaluate the safety and efficacy of FOLFIRI +AFL (EFC11885), grade 3 or 4 neutropenia was occurred in 61.2% of patients (38/61).^33^ These results suggest that the frequency of severe neutropenia in FOLFIRI +AFL treatment in Japan may be higher than those of western countries (i.e., VELOUR trial, 39%).^13^ Thus, confirmation of the UGT1A1 polymorphism that is a determinant of neutropenia^34^, ^35^ is required before considering chemotherapy and supportive treatments, such as granulocyte colony‐stimulating factor, before or during second‐line treatment, especially in FOLFIRI +RAM and AFL. Based on the above results, treatment decisions regarding antiangiogenic drugs should be made according to patient characteristics and background factors.

The results of study were limited because the relatively small number of patients and retrospective study. Randomized clinical trials will be needed to determine the superiority or inferiority of these three drugs. However, it is difficult to perform a prospective comparison of the three drugs. Despite these limitations, the results of this study provided important and novel insights into the clinical use of these drugs and research prospects of second‐line chemotherapy with antiangiogenic agents.

In conclusion, no significant difference in chemotherapeutic efficacy was observed among the three antiangiogenic agents with FOLFIRI. In contrast, we confirmed significant differences in terms of both the frequency and presence of grade 3 or 4 AEs. Grade 3 or 4 AEs within the first four cycles of treatment and prior BEV exposure may be helpful predictor of efficacy in mCRC patients treated with second‐line chemotherapy with VEGF inhibitor.

## CONFLICT OF INTEREST

The authors declare that they have no conflict of interest.

## AUTHOR CONTRIBUTIONS

Hiroki Osumi and Eiji Shinozaki conceptualized and designed the study. Hiroki Osumi, Eiji Shinozaki, and Akira Ooki analyzed and interpreted the data. Hiroki Osumi drafted the manuscript and performed statistical analysis. Eiji Shinozaki, Akira Ooki, Takeru Wakatsuki, Daisaku Kamiimabeppu, Taro Sato, Izuma Nakayama, Mariko Ogura, Daisuke Takahari, Keisho Chin, and Kensei Yamaguchi involved in critical revision of the manuscript for important intellectual content. Eiji Shinozaki and Kensei Yamaguchi involved in study supervision.

## Funding information

This research did not receive any specific grant from funding agencies in the public, commercial, or not‐for‐profit sectors.

## ETHICS APPROVAL AND CONSENT TO PARTICIPATE

All procedures followed were in accordance with the ethical standards of the responsible committee on human experimentation (The Cancer Institute Hospital of Japanese Foundation for Cancer Research, Institutional Review Board, approval number 2020–1017) and with the Helsinki Declaration.

## CONSENT FOR PUBLICATION

The protocol was described on the hospital website and subjects were provided the opportunity to opt out; therefore, no new consent was required from patients.

## Supporting information

Figure S1

Figure S2

Table S1

## Data Availability

This work is licensed under the Creative Commons Attribution 4.0 International License. To view a copy of this license, visit http://creativecommons.org/licenses/by/4.0/.
