# Supplementary material for: Early hypertension and neutropenia are predictors of treatment efficacy in metastatic colorectal cancer patients administered FOLFIRI and vascular endothelial growth factor inhibitors as second‐line chemotherapy
Source: Cancer Med. 2020 Dec 21;10(2):615–25. doi: 10.1002/cam4.3638 (PMC7877370; doi:10.1002/cam4.3638)
Supplement: Supplementary file 1 — Figure S1 [file CAM4-10-615-s001.pdf]

(a) (b)

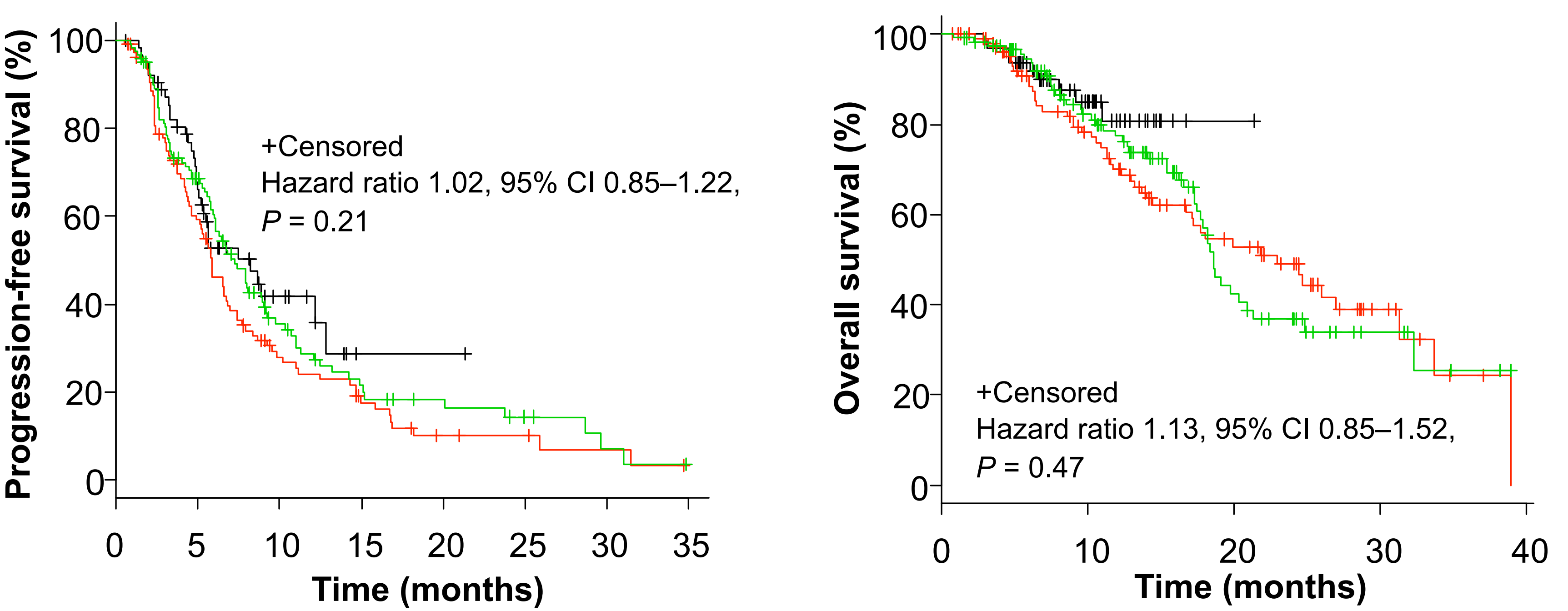

	n	median	95% CI
FOLFIRI+BEV	119	7.2 months	6.0 to 9.0
FOLFIRI+RAM	107	5.8 months	4.6 to 6.8
FOLFIRI+AFL	63	8.2 months	5.2 to 12.8

	n	median	95% CI
FOLFIRI+BEV	119	18.6 months	17.4 to 21.3
FOLFIRI+RAM	107	23.0 months	16.7 to 31.3
FOLFIRI+AFL	63	NA	NA to NA
