# Supplementary material for: Early hypertension and neutropenia are predictors of treatment efficacy in metastatic colorectal cancer patients administered FOLFIRI and vascular endothelial growth factor inhibitors as second‐line chemotherapy
Source: Cancer Med. 2020 Dec 21;10(2):615–25. doi: 10.1002/cam4.3638 (PMC7877370; doi:10.1002/cam4.3638)
Supplement: Supplementary file 2 — Figure S2 [file CAM4-10-615-s002.pdf]

(a) Non prior Bev Group

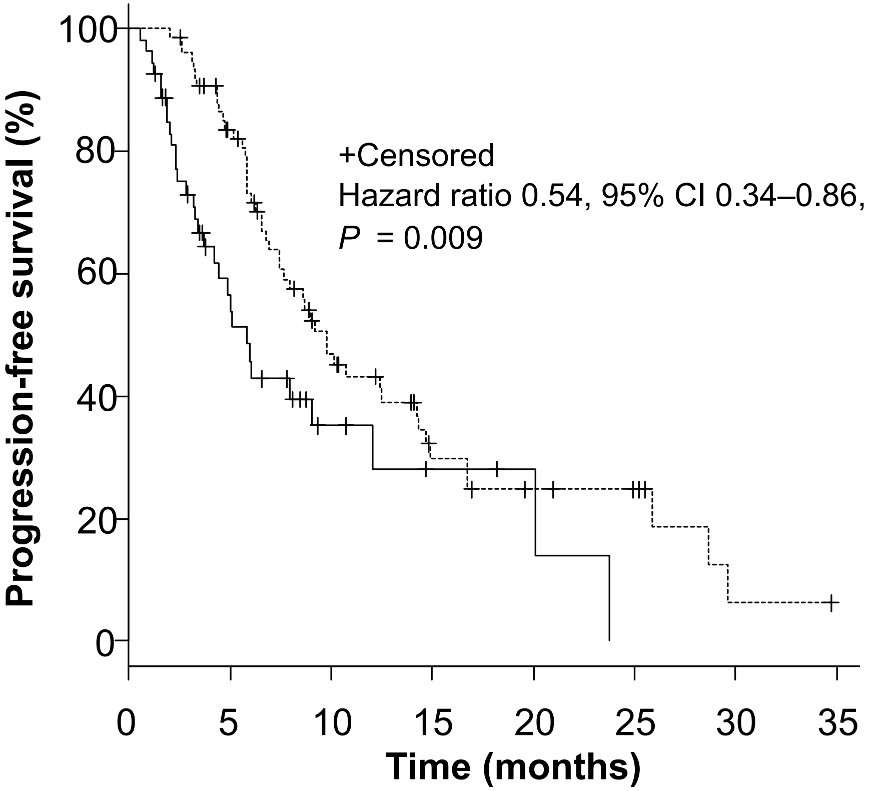

	n	median	95% CI
— G3/4 adverse event (−)	66	5.8 months	3.7 to 12.1
· · · G3/4 adverse event (+)	64	9.8 months	7.4 to 14.2

(b) Prior Bev Group

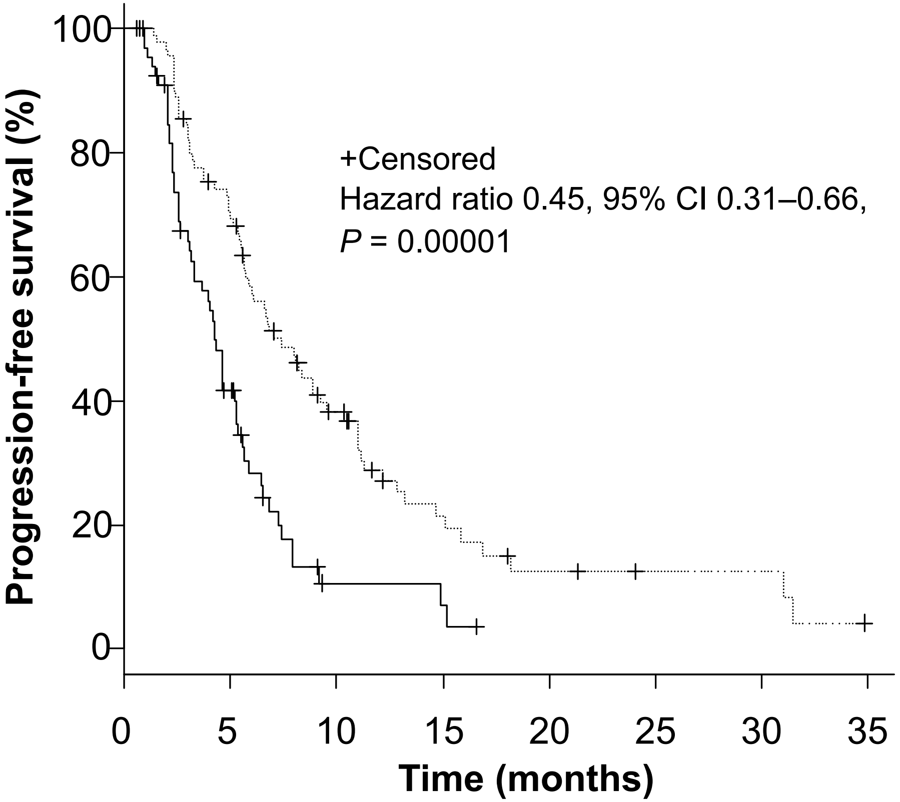

	n	median	95% CI
— G3/4 adverse event (−)	81	4.2 months	3.1 to 5.2
· · · G3/4 adverse event (+)	78	7.1 months	5.6 to 9.2
